# “You left a void that we will never be able to fill”: The legacy of Edmond Amran El Maleh in a contemporary Moroccan novella

**DOI:** 10.12688/openreseurope.16430.1

**Published:** 2023-09-29

**Authors:** Fernanda Fischione

**Affiliations:** 1 SARAS, University of Rome La Sapienza, Rome, Lazio, 00185, Italy

**Keywords:** Moroccan Jews, Maghrebi novel, Edmond Amran El Maleh, Jewish-Muslim relations, postmodern Arabic literature

## Abstract

The sudden depart of a large portion of the Moroccan Jewish population between 1948 and 1967 left a void in Moroccan society, to the extent that some scholars account for the existence of a “double trauma” – a trauma for both those who left for Israel and the Moroccan society at large. This profound social wound has never healed. The Moroccan Jewish intellectual Edmond Amran El Maleh (1917–2010) is the hero of the novella *Aḥǧiyat Idmūn ʿAmrān al-Māliḥ* (The riddle of Edmond Amran El Maleh) by Mohammed Said Hjiouij ( 2020), which this article analyses. In this novella, Hjiouij stages the double trauma of Jewish and Muslim Moroccans by giving voice to the liminal character of El Maleh, a harsh critic of Zionism and French colonial ideology. A metaphor for the marginal writer and a symbol of collective trauma, the figure of El Maleh is re-employed and loaded with new functions and meanings in a contemporary work of fiction with a post-modern aesthetics.

## Jews and Muslims in Morocco: a (hi)story of absence and trauma

In December 2020, the Kingdom of Morocco and the State of Israel signed the Abraham Accords, a set of agreements through which the two countries ratified their decision to openly engage in diplomatic relations, economic cooperation, and cultural exchanges ^2^. In addition, the USA recognised Morocco’s contested sovereignty on Western Sahara, arousing the wrath of many who saw the treaties as an umpteenth violation of human rights and another step towards the erasure of both Western Sahara and Palestine from the map ^3^. However, the Abraham Accords have just made visible and normalised the backroom relationships the two countries have maintained for decades ^4^.

Muslims and Jews in Morocco can claim a long history of coexistence, but what used to be the most populous community of *Mizrahim* – *i.e.,* Oriental Jews ^5^ – is reduced to around 3,000 people today, mainly due to emigration to Israel, France, and Canada, or other countries. What were the consequences for Moroccan society of such massive drainage of people? Besides the historians who have retraced the history of Moroccan Jews from antiquity to the present mainly relying on archival materials ^6^, other scholars ( *e.g.*, Levy, 2001 and Levy, 2003; Trevisan Semi, 2010, and Boum, 2013, among many others) have tried to answer this question from a more subjective or ethnographical point of view, by analysing the way both Muslim Moroccans and Jewish Moroccans or Jews of Moroccan origin perceive this phenomenon. Despite their different stances and perspectives, they all agree that the departure of a significant portion of the Moroccan population was a unique and traumatic historical process since it was sudden, semi-secret, or at least surrounded with mystery, and it “was not mourned or ritualized in any way” ( Trevisan Semi, 2010: 118). In a few years, spanning from the second half of the Forties to the end of the Sixties ( Giardina, 2018: 36–46), centuries of interreligious and intercultural coexistence seemed to be effaced.

This effacement, however, left traces that have never stopped haunting Moroccan society and the Moroccan Jewish diaspora until today. As André Levy highlights, the peculiarity of this effacement challenged the “‘solar system model’ of homeland-diaspora relationships” ( Levy, 2001: 246), allowing room for multiple sites of nostalgia and making the relationship between the homeland and the exilic space more complex and multifaceted than ever. The awkward ghosts of these former fellow countrymen fluctuate above present-day Morocco. To quote Levy (2003: 365–366),

It is as if the Jews continued to exist [in Morocco] but as a shade, a feeble yet lingering national and personal memory. Those Jewish individuals who do remain seem to embody the past. There is an irony here, in that Morocco’s Jewish *absentees* remain present in the landscape, whereas *present-day* Jews appear to be absent.

While we can rely on a rich scholarly literature on the Moroccan Jewry in the 20 ^th^ century that digs into the Jewish archives ( *e.g.*, Laskier, 1983 and Laskier, 1994), and explores the life of these communities both in Morocco and in Israel ( *e.g.*, Benichou Gottreich & Schroeter, 2011; Zohar, 2005), we know less about the perspective of Muslim Moroccans and, more specifically, how they took the evaporation of around 2.7% of the population between the 1950s and the 1960s ( Boum, 2013: 1). Some scholars, however, have recently begun to focus on the subject in their ethnographic works and to understand how the Moroccans’ collective memory deals with it. For instance, by researching the archives of Jewish and Muslim (and mainly Amazigh) families in Southern Morocco and interviewing their members, Aomar Boum (2013) provides a unique insight into the memories of the people who witnessed the disintegration of their communities after the Jews’ departure. Interviewing four generations of male respondents in ʿAqqā, Boum sheds light on how the memory of Jewish-Muslim coexistence has changed over time, influenced by the unfolding of different historical events and processes, from the French Protectorate to the post-Oslo agreements era. As Boum admits, on a few occasions, the local people he met during his fieldwork considered his ethnographic research suspicious. An insider of the communities he was studying, he felt his position was awkward. Moreover, his interviewees found it weird that a native Muslim anthropologist working for a US university was researching Southern Moroccan Jews. This anecdote is symptomatic of what Boum describes as an actual taboo surrounding the Jewish presence-absence in Morocco.

An open discussion on the fate of the Moroccan Jewish community would be relevant for the local society to process the trauma and build a more equal and plural community. However, the relatively sudden disappearance of thousands of Moroccans did not leave any trace in the public debate for many years. As Trevisan Semi (2010: 122–123) highlights, the traumatic, painful, and unexpected departure of one of the historical components of Moroccan society has long been denied and concealed. It neither sparked a debate on the status of minorities within Moroccan society nor stimulated a discussion around the integration of the Jews in a new Arab nation-state. Moroccan Muslims – according to Trevisan Semi – buried the trauma caused by the Jews’ departure under thick layers of denial and discharge of responsibility. Such denial was also possible thanks to conspiracy theories, according to which the Jews did not flee the country by their own will, but the Mossad forced them to do so ^7^.

Jews in Morocco have a long history, dating back to the pre-Islamic period (4th Century B.C.). Before World War II, at its peak, the community counted around 240,000 people (2.7% of the total population), most of them living in the main urban centres of the Kingdom but also in the Southern regions of the Anti-Atlas Mountains. Despite sometimes facing distancing (above all in urban environments) and discrimination and occupying a weak position within the country’s political and demographical balance, Moroccan Jews lived relatively well compared to European Jews. As Boum (2013: 15) shows, for example, the social and economic ties between the Amazigh tribes of the South and their Jewish protégés were so tight that only a small number of Moroccan Jews of the Bled (namely, the hinterland) applied for the French protégé-status from 1933 onwards, being afraid of losing the protection of local tribes.

Things changed with the arrival of the Alliance Israelite Universelle (founded – among others – by Adolphe Crémieux, the proponent of the 1870 Crémieux Decree in Algeria), which took over the affairs of local Jewish communities in Morocco, providing them with schooling and other services. Since then, the destiny of Moroccan Jews has become more closely linked with Zionism and Israel ^8^. Following the Zionist propaganda, Moroccan Jews began to emigrate to Israel upon the foundation of the State, but in 1958 independent Morocco adhered to the Arab League and cut ties with Israel, curbing the emigration of Moroccan Jews ( Abitbol, 2014: 666). Emigration could not be stopped, though: between 1956 and 1961, the Mossad smuggled over 12,000 Moroccan Jews to Israel, followed by another 80,000 between 1961 and 1964 ( Laskier, 1989).

Despite all the controversial aspects regarding this “internal other” to Moroccan society, the Jewish component is recognised as constitutive of Moroccan identity at large, as the Preamble to the 2011 Constitution reads:

A sovereign Muslim State, attached to its national unity and to its territorial integrity, the Kingdom of Morocco intends to preserve, in its plentitude and its diversity, its one and indivisible national identity. Its unity, is forged by the convergence of its Arab-Islamist, Berber [amazighe] and Saharan-Hassanic [saharo-hassanie] components, nourished and enriched by its African, Andalusian, Hebraic and Mediterranean influences [affluents] ^9^.

The enshrinement of a Jewish component into the constitution is only the culmination of a long-lasting process aimed to reformulate a multifaceted Moroccan identity able to cope with the trials of the 21 ^st^ century. As Emily Gottreich (2020: 3) highlights, “these days it is rare to open a newspaper or watch Moroccan television without seeing a ‘Jewish’ story”, and the Jewish past of Morocco constantly resurfaces in the form of freshly rebuilt synagogues, restored cemeteries and saints’ tombs, museums and other public-space landmarks.

If the reasons for such interest on an institutional level are to be found in the internal and external challenges the Sharifian Kingdom must address – and especially the necessity to counter radical Islam and strengthen its position on the geopolitical arena – cultural actors show that the interest in unpacking and reviving the multireligious history of the country is deep and authentic, as I will highlight in the next section.

### Reconsidering the multicultural past of the Maghreb in fiction

As mentioned above, scholars have only recently begun to shed light on the subjective memories of Moroccan Jews and Muslims. Surprisingly, despite what Trevisan Semi and others argue about the collective undoing of these painful and socially disruptive memories, a growing number of works of fiction published in the last few years in Morocco deal with the relationship between Muslims and Jews and the history of Moroccan Jewry.

In an interview with the newspaper *al-Quds*, however, Muḥammad Saʿīd Iḥǧīwiǧ (from now on, Mohammed Said Hjiouij) – author of the novella I analyse in this article – highlights how it is not easy for potential readers to find such works:

When I was looking for Moroccan novels dealing with the theme of Judaism, I only found two novels, most probably because of the poor capillarity of the distribution. One was penned by a young writer, and, despite its weaknesses, it had some hidden strengths waiting to pop up. The author of the other novel was a famous writer, but I could not read more than a few pages because of his mediocre prose and superficial thinking ^10^.

Besides his considerations on the aesthetic value of the two novels, it is interesting to note how the writer constantly addresses the problems that affect the Moroccan book industry both in his fiction and his epitexts, as I will explain later.

Reflecting on the surfacing of Moroccan Jewry in recent works of fiction, Brahim El Guabli frames such works within what he calls “other-archives”, namely “texts, artifacts, alphabets, embodied experiences, toponymies, and inherited memories where stories of the excluded, the silenced, and the forgotten live in a ghostly state, ready to articulate loss even as they are situated outside the margins of what is considered canonical” ( El Guabli, 2023: 1).

Unlike official, top-down archives, other-archives consider not only what is there but also what is missing, as loss and absence are inherent to their essence. This aspect is crucial to affirm the right to the memory of neglected subjects invisibilised by the official narratives and, subsequently, to expand and democratise the archive concept. El Guabli places the novels tackling the issue of Moroccan Jews under the category of mnemonic literature, which “unlike historical fiction, addresses a situation in which both absence and silence are the norm” ( El Guabli, 2023: 63). Among them, he includes novels in both Arabic and French, such as *Le captif de Mabrouka* (Mabrouka’s Captive, 2009) by Hassane Aït Moh, *Šāmma aw Štirīt* (Šāmma or Štirīt, 2013) by Ibrāhīm Ḥarīrī, *Anā al-mansī* (I Am the Forgotten, 2015) by Muḥammad ʿIzz al-Dīn al-Tāzī, *Zaġārīd al-mawt* (Ululations of Death, 2015) by ʿAbd al-Karīm al-Ǧuwayṭī, *Kāzānfā* (Casanfa, 2016) by Idrīs al-Milyānī, and *Sīntrā* (Cintra, 2016) by Hasan Awrīd. To these titles, I add the short story *Tawārīḫ ummī* (The Stories of My Mother, 2005) by ʿAbd al-Munʿim al-Šantūf, the historical novel *Asfār Yaʿqūb al-arbʿa* (The Four Books of Jacob, 2017) by Ḥasan Riyāḍ, and the novella *Aḥǧiyat Idmūn ʿAmrān al-Māliḥ* (The riddle of Edmond Amran El Maleh) by Mohammed Said Hjiouij ( 2020).

It is interesting to remark that, in the past few years, Maghrebi fiction in Arabic has been reconsidering the legacy of an authentically multicultural and multireligious past, based on a long history of common fears and threats of cohabitation, but also peaceful coexistence and positive interactions among Muslims and Jews.

Pluralism was particularly meaningful in the postcolonial intellectual debate in Morocco, Algeria, and Tunisia (see, *e.g.*, Aoudjit, 2015: 92; Marzouki, 2014: 55). It was an antidote for the nationalist discourses prevailing at that time, whose authoritarian, violent, and one-dimensional features were already visible the day after the Maghrebi states achieved independence. Several Maghrebi intellectuals such as Abdelkébir Khatibi, Edmond Amran El Maleh, Mouloud Mammeri, Kateb Yacine, Albert Memmi, and many others deeply reflected on the idea of a plural society able to value and include its whole multifaceted heritage ( Harrison, 2015). “The Maghreb as a horizon of thought” – as Khatibi called it in an article that appeared in a special issue of Sartre’s *Les Temps Modernes* in 1977 – was supposed to counter a monocultural and authoritarian idea of the nation-State, as it was the one conveyed by pan-Arabism and imposed on the post-independence Maghrebi states through vigorous projects of Arabisation ^11^. According to Khatibi (1977), the Maghreb itself represents a blank space to rethink, starting from the periphery and not the centre, from the difference and not the uniformity. As Idriss Jebari (2018: 58) puts it, “the Maghreb is a name for the ‘unthought’ margin, a space of generative pluralism, and an opportunity for a radical subversion of the limits placed by centralising force and metaphysics of the nation-state”.

### An emergent Moroccan writer and a postmodern novella

Mohammed Said Hjiouij was born in 1982 in Tangier, the city where he still resides and which provides the setting for most of his fictional works. In 2004 he founded the monthly literary magazine *Ṭanǧa al-adabiyya* (Literary Tangier), which was issued for a couple of years, although sporadically. Hjiouij debuted with the short story collection *Ašyāʾ taḥduṯ* (Things that Happen, 2004), followed by another short story collection titled *Intiḥār murǧaʾ* (Postponed Suicide, 2006). After a stop of more than ten years due to his career in the field of blogging and technology, he made his comeback in 2019 with the short novel *Layl Ṭanǧa* (In Tangier by Night), awarded with the Ismaʿīl Fahd Ismaʿīl Prize in Kuwait and followed by another short novel titled *Kāfkā fī Ṭanǧa* (Kafka in Tangier, 2019). In 2020, Hjiouij issued *Aḥǧiyat Idmūn ʿAmrān al-Māliḥ*, the novella I will analyse in this article. Finally, *Sāʿī al-barīd lā yaʿrif al-ʿunwān* (The Postman Does Not Know the Address) – a short novel exploring the world of international espionage and the 1973 Lillehammer affair in particular – came out in November 2022.

Despite its brevity, *Aḥǧiyat Idmūn ʿAmrān al-Māliḥ* has a complex and multi-layered plot, which holds together several sub-plots, styles and literary genres, following a postmodern fragmentary aesthetics remindful of the experimental Arabic prose of the 1960s and the 1970s. Hjiouij defends his style choices quite polemically, stressing how experimental writing is still marginalised in the field of Arabic literature despite its long tradition:

My novels feature some postmodern elements I have purposedly planned to use, while others – such as metafiction and the intertwining of reality, fantasy, and different levels of consciousness – appear unintentionally and effortlessly. […] In the last two years, I discovered Arabic novels from the 1960s and 1970s displaying many postmodern features. I found them mind-blowing. How is it possible that such experimental novels have been there for decades and, nonetheless, the Arabic novel has not developed yet, remaining frozen in a quite classical style? How is it possible that readers and writers still consider Naǧīb Maḥfūẓ the emblem of the Arabic novel? ^12^

Narrated by a third-person narrator who embodies death, the novella’s plot revolves around two main characters – one historical and one fictional – named Edmond Amran El Maleh and Franz Goldstein. The character of El Maleh is inspired by the homonymous, well-known Moroccan Jewish leftist intellectual born in Asfi in 1917 ^13^. His family was originally from the Southern region of Sous and belonged to those “berberised” Jewish tribes that Aomar Boum describes in his *Memories of Absence* (2013). As the novella highlights, the Moroccan political police persecuted El Maleh due to his activity within the banned communist party, his criticism against Hassan II, and the repression the latter unleashed in the Rif (p. 37). In 1965, when he was accused of instigating the revolt of Casablanca, his life in Morocco became impossible, and he was forced to expatriate. He moved to France, where he started writing fiction at a quite old age and worked as a journalist and a professor of philosophy. In 2000 he moved back to Morocco and settled down in Rabat, where he died ten years later ( Keil-Sagawe, 2011).

Conversely, Franz Goldstein – a French Jew of German origin – is a fictional character acting as an Ashkenazi counterpart to the Mizrahi Edmond. One of the narrative functions of Franz’s character is to help the differences between the two Jewish communities emerge, especially concerning the alleged antisemitism of the Arabs and the Jews’ (dis)identification with the Zionist ideology.

There is much in common between Amran, the hero of the novella, and the actual Edmond Amran El Maleh: both fled Morocco for France; both are journalists; both vocally oppose Zionism. The fictional Amran is portrayed as a journalist working for the books section of *Le Monde* and writing a novel that he is struggling to complete. Moreover, he is a jury member of an independent literary prize. At the beginning of the story, he meets with Franz Goldstein in a café in France. Goldstein, who owns and manages a publishing house named Éditions de Sable, wants to convince Amran to award the prize to a novel titled *The Holy Day* and published by his company. Attempting to corrupt Amran, Goldstein offers him a good amount of money, promising to publish his novel once he finishes writing it and reward him with an extra sum. Amran is reluctant and initially refuses. In the end, however, Goldstein succeeds in his attempt since Amran takes the money. Nevertheless, Amran eventually boycotts the conspiracy orchestrated by Goldstein, who begins to persecute and threaten him for not sticking to their agreement.

At the end of the novel, the plot gets less and less structured, and dreams and reality become almost indistinguishable until Amran ends up locked in a psychiatric hospital in Casablanca, forgetful of his identity and trying to recollect his vanishing memories. Individual memory is perhaps unreliable, as El Maleh’s fragmentary and contradictory reminiscences seem to warn us at several points of the novella, but recording it as fictional writing is sometimes the only way to resist “to the amnesia fostered by official history” ( Vogl, 2003: 77). By embracing this point of view, Hjiouij departs from the grand narratives of the historical novel and allows a more subjective, plural and fragile understanding of memory.

Around this main storyline, at least three other sub-plots unfold: one concerns Amran’s past and his reflections about his plural identity; another one revolves around the imaginary character of Aunt Maymūna, Amran’s old aunt, who appears in his dreams and tells him old stories drawn from the Moroccan (Jewish and Amazigh) folkloric heritage; and the last one is a nightmarish stream of consciousness unravelling as Amran progressively loses his sanity. The novella also features some metafictional reflections, ranging from the material process of writing to the corruption of the book industry and the downsides of literary prizes, as I will highlight in the following section.

Despite its short length, *Aḥǧiyat Idmūn ʿAmrān al-Māliḥ* mixes many styles, moods, and sub-genres using a pastiche technique. For instance, as the story retraces the history of Moroccan Jews and the narrator mentions historical events, dates, names, and figures, the style and content are close to those of an essay (for example, p. 27–28). Nonetheless, from time to time, the reader finds “stories within the story”, shaped either as dreams, stories of magical realism, or tales drawn from the Amazigh repertoire, such as those collected by Émile Laoust ^14^. Moreover, the narrative pact between the author and the reader is constantly broken. For example, when the reader is convinced that he is reading a historical account of El Maleh’s vicissitudes, his belief is shaken by nightmarish scenes where Amran is portrayed as a madman in the hospital who does not remember whether the episodes of his life he has previously narrated did indeed happened or not.

The reader also finds a lengthy summary of the novel Amran is writing (p. 29–33), an apocalyptic dystopia inspired by Margaret Atwood’s *The Handmaid’s Tale* and titled *Barzaḫ al-ḥikāyāt* (The Limbo of Tales). Moreover, in the second part of the novel, Franz Goldstein tells the story of the killing of his family, and the narration changes to horror and pulp.

### Therapeutic history and the Moroccan Jew as a metaphor of the marginal writer

In an interview I had with him recently, Mohammed Said Hjiouij said that in his novella *Aḥǧiyat Idmūn ʿAmrān al-Māliḥ*, he wanted to explore the topic of the emigration of the Moroccan Jews and especially the secret operations through which the departures were arranged (see underlying data):

I wanted to write about the ship Egoz, which Mossad employed in its secret operations to move the Jews from Morocco to Gibraltar and from there to Israel. On January 11th, 1961, on its thirteenth journey, the ship sank along with its 44 Jewish passengers, half of whom were children ^15^.

The sinking of the Egoz was a turning point in the life of Moroccan Jews, marking the beginning of systematic, secret cooperation between Israel and Morocco. From that moment onwards, it became clear that it was not possible anymore to leave emigration in the hands of the smugglers working for the Misgeret (an emigration agency established by Mossad). As a result, King Hassan II secretly ratified Operation Yakhin, letting 50,000 Jews leave Morocco between 1961 and 1964 ( Giardina, 2018: 43–44). Not only was the Moroccan State aware of the intense (and officially illegal) displacement of a part of its population, thus, but it also received money from the Hebrew Immigrant Aid Society as compensation for the administrative costs. By 1964, Morocco counted less than 80,000 Jews, decreasing in number until they reached the meagre figures of today ( Giardina, 2018: 44).

Telling this story today has both educational value and a critical function, especially when the media and the world of politics enthusiastically celebrate the normalisation of the relations between Israel and Morocco. By recalling such dark episodes from the history of Morocco and especially from Hassan II’s reign, Hjiouij acts as a helpful killjoy. Leaving aside easy enthusiasm, in fact, he warns us against the danger of forgetfulness and reminds us that the history of Moroccan-Israeli relations has gone through a painful and deadly secret path before resulting in today’s open agreements. 

Preserving the memory of Muslim-Jewish coexistence in Morocco is much needed today. For various reasons, the transitional justice and reconciliation process that King Mohammed VI launched at the beginning of his reign does not include the question of Moroccan Jews. Despite all the predicaments they went through, the latter were able to exert their agency and were not powerless victims ( Trevisan Semi, 2010). They sold their goods and fled the country willingly, confident they could rely on Israeli aid and a vast network of transnational institutions ( Bin-Nun, 2014: 204). However, there is still an awkward aura of suspension surrounding their disappearance. In her analysis of 1986 El Maleh’s novel *Mille ans, un jour*, Ronnie Scharfman (1993: 140) argues that

The politically motivated emigration of the Moroccan-Jewish population is only metaphorically an ethnocide, of course, […], but in the universe of this novel, its inscription on the body of the exiled Jew is as indelible and as violently wrought as the Nazi’s tattoo or the slave’s brand. The departure, en masse, of this community has left a void that can never be filled, and the consequences for El Maleh are that he textualizes this exodus as, ironically, an exile to “The Promised Land”.

Therefore, if not the State reconciliation procedures, literature – as a form of “therapeutic history” ( Jebari, 2018) – can at least track such an uncomfortable feeling of loss and the collective trauma that caused it. “What remains untold in the national archives has been voiced through a collective process of memorialisation”, as Cristina Dozio (2024) puts it, and literary fiction is a privileged site for such a process to unfold.

Mohammed Said Hjiouij chose Edmond Amran El Maleh as the protagonist of his short novel for several reasons ^16^, the first of which has to do with the writer’s fictional needs:

The hero of a novel about the Jews must be Jewish. I did not know about any other Jewish personality except Edmond Amran El Maleh, an opponent of the Zionist movement (and the State of Israel itself) and a political dissident of Hassan II’s regime. […] However, I departed from the well-known clarity of El Maleh, and I depicted him as someone who constantly has doubts and questions, even about his own memories ^17^.

Another reason is more closely related to sociological issues that run through the whole novella and concern the status and position of the writer. Through El Maleh’s character, in fact, Hjiouij also wanted to explore the marginal position of young Moroccan writers today:

Another feature of El Maleh is his clashes with the French publishers over his literary choices and the marginalisation he went through due to his critical stance towards Israel. This is a crucial aspect of his persona. It is also an issue that involves me personally since I live on the margin and observe the corruption of literature from a distance. That is why I did not find any difficulty in mixing the history of Moroccan Jews in Israel and the moral decline of the cultural environment and the literary prizes ^18^.

Franz Goldstein – a character embodying the evils of Zionism, Israeli colonialism, and the mafia in the book industry at once – represents the corruption of the literary prizes and the publishing sector, with which the writer polemises.

As his production makes clear, Hjiouij specialises in short fictional formats, an aspect which he embraces as a crucial part of the aesthetics and politics of his writing. As he declares in an interview with Yāsīn ʿAdnān (Yassine Adnan),

We must acknowledge that the length [of a novel] is not everything. We need literary prizes to take into account the category of the novella or even new awards where novellas only are listed. I feel frustrated when I am told that one of the conditions a novel must satisfy to compete in a literary prize is a length of at least 25.000 words. It is even more frustrating that the Ismaʿīl Fahd Ismaʿīl Prize for the short novel has been cancelled this year, apparently for good ^19^.

The influence of literary awards on Moroccan (and Maghrebi) literature in Arabic is quite heavy since awards are one of the very few channels through which such literature can be circulated both within the Arabic-speaking region and worldwide in English translation and grant its authors recognition, readership, and material gains. Opening up to the global markets is vital for young writers who do not have sufficient social and economic capital within their respective national fields. Moreover, local book industries in the Maghreb are hindered by a systematic lack of structure, piracy, and underinvestment ( Pickford, 2016: 80–81). As Pickford (2016) highlights, despite a gradual improvement of the situation over the years, Maghrebi literature still represents a niche within the niche of Arabic literature in the anglophone book industry and academia. While francophone literature from the Maghreb benefits from its commodification as a “postcolonial [good] produced for French readers” ( Laroussi, 2003: 88) and has partially gained a place in the Parisian literary establishment, Maghrebi literature in Arabic still suffers from a great degree of marginalisation. Thus, Maghrebi authors writing in Arabic have essentially two ways to try to reach the anglophone market: either they are awarded (or at least shortlisted for) a prestigious international prize, such as the IPAF, or they get consecrated through French translation and eventually make it to the UK-US market. In both cases, the writers can hope to succeed only “as long as they wrote in accordance with reader expectations” ( Pickford, 2016: 87).

Reluctant to purposely adopt a specific novel format and a writing style only to please the public, Hjiouij experimented with an original translation and distribution plan for his 2019 novel *Kāfkā fī Ṭanǧa*. US translator Phoebe Bay Carter translated the novel into English, and the unedited draft translation was distributed in weekly instalments through a newsletter to which anyone could subscribe on the author’s website. The goal of the project was to find a publisher interested in editing and commercialising the novel while at the same time trying to widen the distribution network and the author’s readership.

The interest in reaching the anglophone readership also lies in the rising importance of English among the younger generations. According to a survey by the British Council (2021: 6),

young Moroccans believe it is more important to learn English than Arabic or French. 40% of respondents believe English is the most important language to learn, compared with only 10% for French. English is considered slightly more important to learn than Arabic, with 65% and 62% respectively believing each language is either the most important or an important one to learn.

Finally, the non-neutral ideological status of French in today’s Morocco should also be considered when attempting to understand why English seems to be a more appealing language to be translated into. When I asked Hjiouij whether he was interested in entering the francophone field, he answered that

To me and my generation, French represents a colonial language. By colonial, I do not mean the military occupation our grandfathers suffered, but the economic colonialism represented by the control that certain Moroccan families exert over the local economy by collaborating with some French enterprises. It also gives me a feeling of alienation since French is the language of administration and economy despite Arabic being the country’s official language. It is as if I was not in my own country. […] French in Morocco represents corruption in all fields – administrative, political, and economic. It represents all my stolen rights as a Moroccan citizen ^20^.

According to Hjiouij, French is the language of the older generations. Therefore, it does not mean much to the young writers today, who have been exposed to the US media through the Internet and have found a chance to set themselves free from what he calls “the prison of French ‘excellence’”.

## The Moroccan Jew as the other within: double critique and disidentification

Interestingly, Amran and Franz – the story’s two main characters – represent two sides of the Jewish identity. Edmond Amran El Maleh is a Moroccan Jew who did not experience the Holocaust and had the chance to observe Israel’s birth and development from a relatively privileged standpoint – a standpoint of “double critique”, to name it with Abdelkebir Khatibi. According to Khatibi (2019: 26), double critique is a mission of Arab sociology consisting of two tasks:

1. a deconstruction of logocentrism and ethnocentrism, this speech of self-sufficiency par excellence that the West, in the course of its development, developed on the world […].
  2. […] a critique of the knowledge and discourses elaborated by different societies of the Arab world about themselves.

Double critique addresses a society’s outside and the inside, but it does it in a non-oppositional and non-essentialist way. The Jew is not “the Other” as opposed to “the Self”: it is the Other within the Self. The “privilege” El Maleh enjoyed was this liminal position between worlds, implying both the fragility and the strength of those who embrace it. On the one hand, El Maleh was an insider of the Jewish community and was aware of the transnational ties that bounded Moroccan Jews living in Morocco and Moroccan Jews living in Israel. On the other hand, as a Moroccan Jew, he feels part of another history that has little to do with the history of the Jews from Central and Eastern Europe.

Much of El Maleh’s intellectual effort was devoted to “disproving the myth of a timeless and intrinsic Jewish-Muslim/Arab enmity” ( Harrison, 2015: 129). Moreover, according to Harrison, in his works of fiction – especially *Mille ans, un jour* – El Maleh attempted to draw a comparison between the oppression of Palestinians and the oppression of Arab Jews who fled their countries and emigrated to Israel just to find themselves uprooted, discriminated, and forced to live in miserable conditions. Israel, thus, produces colonial injustice not only against the Muslims but also against the Jews that it claims to protect. Israel has adopted discriminatory policies towards the Jewish communities of the Maghreb since its foundation. As the journalist Arieh Gelblum wrote about Mizrahi Jews in 1949, less than a year after the establishment of the state of Israel: “from the standpoint of their primitiveness, their level of education and their ability to absorb anything spiritual, [the Maghrebian Jews] are even worse than the Arabs in Palestine” ( Hochberg, 2007: 95). And as the fictional El Maleh observes:

The pride of Israel nestled in my soul […] began to fade away on the very first day of my arrival in Israel when I found myself in a rigid religious school. I also found myself obliged to work in agriculture instead of [embracing] the future I had been expecting, the future I was awaiting and hoping and craving for, in the world of culture and journalism. (p. 13–14)

In the novel, Amran seems to be disidentified with both Israel and Palestine because he does not recognise the necessity of building an identity-based homeland for the Jews anymore:

Before moving to Israel, I remember being sympathetic toward the Palestinians and understanding their right to defend their land. Still, at the same time, I sensed that Israel was my land and I was ready to defend it from the Arab army. However, I have changed my mind in front of these blank pages on which my pen moves as fluid as a stream of consciousness. I am not supportive of the Palestinians anymore (how could I be supportive of the remnants of a people torn apart by internal fights for a delusive power?), and I do not consider Israel my land anymore, nor do I see the necessity of having a special State for the Jews. But, at the same time, I understand the right of the Sabra Jews [ *i.e.,* the Jews living in Palestine before the foundation of Israel] to find a home in the land they live in, whether they call it Palestine or Israel. (p. 23)

Furthermore, caught between his Moroccanness and his Jewishness, Amran adds:

I confess that I used to consider myself Jewish in the first place and Moroccan in the second or third place for years. But now I hate this expression that Franz keeps repeating: *al-yahūd al-maġāriba* [Jewish Moroccans]. What is wrong with *al-maġāriba al-yahūd* [Moroccan Jews]? (p. 26)

These words summarise the argument El Maleh made in his article “Juifs marocains et marocains juifs” ( El Maleh, 1977). Here, he prioritises belonging to Morocco – which, in his case, is not an abstract belonging but a concrete one, linked to places and human relations – over belonging to Israel – an abstract community where the place for Mizrahi Jews is already set.

However, Amran knows that Morocco could not be a permanent land for him and the other Moroccan Jews since he is “the internal Other”. He recalls an episode of his youth, for example, when he was beaten and humiliated by some Muslim guys who found him guilty of asking for the hand of Iman, a Muslim girl he fell in love with (p. 34–35). Amran is stuck in a paradoxical position where his identities crosscut each other, and he cannot choose one over another. Wondering which land he sees for himself, Amran asks:

Is it Morocco, although I had known for a long time that it only represented a temporary phase, a gateway to France after the diploma? Or is it Israel, which attracted us out of pride after its victory at the Six Days war and, in a few years, closed upon us like a prison full of corruption and greed? We discovered that we were only numbers useful for the elections over there, numbers that the political parties competed for during the elections and eventually threw away.
  We had always known that our presence in Morocco was temporary and that upon our graduation, we would move out and never return. Not necessarily to Israel, about which we did not know anything then, but also to France, of which we spoke the language and in whose schools we used to study together with French students. (p. 44)

Unlike Amran and as a German Jew, Franz has a different standpoint on these issues: he thinks that being a Jew like him, Amran must have gone through the same antisemitism and that the Muslims have a big responsibility in the depart of the Oriental Jews. However, as a European Jew, Franz has a different life experience than Edmond. He publicly presents himself as a survivor of the Holocaust whose family was exterminated in Auschwitz and has also written a memoir about this experience. However, in one of those nightmarish subplots scattered in the novella, Franz eventually confesses to Amran that he killed his whole family and made up his life story. He has never been in a concentration camp, and he tattooed the number on his wrist by himself. Before escaping to France, he poisoned his sisters, knocked out his mother, and set fire to the house. As far as his abusive and alcoholic father is concerned, Franz confesses that he had provided him with some fake documents to let him leave Germany, which was forbidden for the Jews at that time, and had reported him to the Gestapo so that he got arrested.

Franz’s belief in the antisemitism of the Arabs is the opposite of El Maleh’s stances about this issue. In his “Juifs marocains et marocains juifs”, El Maleh (1977: 497) states:

Whenever – willing or not – a Jew emigrates, public opinion immediately raises a fuss about the existence of antisemitism. According to it, it is a self-evident truth, a universally recognised postulate that does not require any demonstration. No one would dare question that history has known tragic persecutions of which the Jews were the victims, genocides as is the case for Hitler’s Germany. However, the scandal starts when, in the name of something that truly happened in some specific historical conditions, we mix everything up and, behind that mask, allow blatantly ideological manipulations.

The objective of the manipulations, concludes El Maleh, is “to justify the existence of the State of Israel and its annexationism and imperialism” ( El Maleh, 1977: 497).

The novel also contains a third Jewish character, briefly sketched on page 43. It is Šimʿūn Dankūr, a fictional Iraqi Jew who ran away from the persecution the Baath Party unleashed against him due to a trivial love skirmish between him and an official of the army. Šimʿūn, a friend of El Maleh, is a professor of Arabic language and a passionate admirer of the poet Badr Šākir al-Sayyāb, whose poem *Unšudat al-maṭar* (The rain song, 1960) he knows by heart. Šimʿūn highlights the complexity of Jewries’ mosaic and touches upon the language question, a particularly thorny issue for Hjiouij’s El Maleh. The latter is fluent in Moroccan colloquial Arabic thanks to his mother and owes his knowledge of French to his father, who “considered French the language of sophistication and civilisation and looked like a Frenchman in all his details: his clothes, food, drinks” (p. 38). El Maleh wonders whether the Jewish community has contributed to its own isolation within Moroccan society:

Now that I think about the language issue, I reflect on the fragility of our peaceful coexistence with the Muslims and how we contributed to this situation ourselves. How can we expect our Muslim neighbours to trust us when we do not attend their schools or speak their language? Maybe it has not always been like this, though. Maybe they ousted us from their schools. (p. 38)

Šimʿūn is also an allusion to the Arabisation process of the Maghreb, which took place starting in 1958 when the Moroccan government called several teachers from Syria, Egypt, Jordan, and Iraq to teach Arabic in Moroccan schools ( Grandguillaume, 1983: 71). As some testimonies highlight ( Levy, 2003: 373; Trevisan Semi, 2010: 115), such a process was traumatic for the Jews. The latter used to receive French education in Moroccan lycées and suddenly found themselves forced to switch to a language and a curriculum they felt alien to the Moroccan culture. Arabisation increased the feeling of isolation of Moroccan Jews while forcing Moroccan culture at large into a monolingual cage and jeopardising its diversity.

Nevertheless, as its members became fewer and fewer, the Jewish community increasingly shut itself off from the outside world. Scholars have shown that the progressive isolation the Jews went through was a result of both Arabisation and the centralising policies of community institutions such as the Jewish Community Council, active in Casablanca. As André Levy (2003: 373) stresses,

the community’s educational system actively contributes to this isolation by ignoring the instruction of Arabic. […] it is quite striking that practically all Jews (except a tiny layer of intelligentsia) do not comprehend the news on national TV […]. Practically all Jews do not speak, let alone read, fusha (“classical” Arabic) and most Jewish youth speak darija (indigenous “dialect”) poorly.

Moreover, Moroccan Jews living in Israel also seem doomed to aphasia, as the fictional El Maleh notices. When he visits Israel, he finds that Moroccan immigrants have reconstructed their original village communities there, where they are once more forced to speak a “foreign” language (Moroccan Arabic) since they do not know Hebrew (p. 38).

## Conclusion

In 2016, Houria Bouteldja, a French-Algerian political activist from the Parti des Indigènes de la République (PIR), published a pamphlet titled *Les Blancs, les Juifs et nous. Vers une politique de l’amour révolutionnaire*. In the third chapter of this brief essay, characterised by harsh and passionate tones, Bouteldja (2016: 59–60) acts as a spokesperson for the Arabs and addresses the Jews with the following words:

Anti-Semitism is European. It is a product of modernity. […] It has confined you to the lower echelons of the hierarchy of honors, but it is not universal. It is circumscribed in space and time. […] You who are Sephardic, you can’t act as though the Crémieux Decree hadn’t existed. You can’t ignore the fact that France made you French to tear you away from us, from your land, from your Arab-Berber identity. If I dare say so, from your Islamic identity. Just as we have been dispossessed of you. If I dare say so, of our Jewish identity. Incidentally, I can’t think about North Africa without missing you. You left a void that we will never be able to fill, and for that I am inconsolable. Your alterity becomes more pronounced and your memory fades.

According to Bouteldja, the Jewish-Muslim enmity is a colonial invention that sowed discord between two communities that could live together before the French and the Zionists disrupted their coexistence. In his “Juifs marocains et marocains juifs”, which I mentioned above, El Maleh vehemently opposes Albert Memmi’s position on Israel, according to which Zionism is an anticolonial liberation movement, and claims that “no Arab country […] has ever known such an extermination policy as Germany’s” ( El Maleh, 1977: 498). Several years apart, El Maleh and Bouteldja – a Moroccan Jewish man and an Algerian Muslim woman – call for the deconstruction of the allegedly universal category of antisemitism and the acknowledgement of the colonial nature of the wound left by the emigration of the Jews from North Africa.

As I have highlighted in this article, the novella *Aḥǧiyat Idmūn ʿAmrān al-Māliḥ* repurposes the figure of El Maleh as a metaphor of the young writer’s marginality in Morocco and a catalyst for the collective debate about the Maghreb’s plural identities. Author Mohammed Said Hjiouij embraces a counterhegemonic aesthetic recalling the experimental Arabic novel in the 1960s and 1970s, going against the grain of commercial Arabic literature and preferring the fragmentary and tentative style of postmodern fiction over the grand narratives of historical novels. By making such a choice, Hjiouij deliberately devotes himself to minor literature, a site whose “cramped space forces each individual intrigue to connect immediately to politics” ( Deleuze & Guattari, 1986: 17). In my interview with him, Hjiouij expressed this imbrication of the individual and the political by declaring that he did not “want to write a boring novel presenting an important question. The challenge was to attempt to write a ‘personal’ novel mixed with a ‘plot’ novel. To combine a novel loaded with profound intellectuality while featuring dynamic and exciting events”.

While rejecting the stiffness of engaged narratives, Hjiouj draws the reader’s attention to crucial issues such as the corruption of the publishing industry, the marginalisation of the intellectuals who propose counternarratives, and the problematic disappearance of the Moroccan Jews.

The question of Maghrebi Jews in contemporary Arabic fiction is still understudied and deserves to be explored more in-depth. Although limited in scope, this article is just a first step towards this objective.

## Ethics and consent

The author has received written informed consent for the individual to be identified by name as the focus of this study and for any data from the interview conducted to be published.

## Data Availability

All data and materials are available on Zenodo. DOI https://doi.org/10.5281/zenodo.7339646. This project contains the following underlying data: أسئلة فرناندا.docx. (consisting of the interview with the author conducted on 07/10/2022.) Data are available under the terms of the Creative Commons Attribution 4.0 International Public License.
