# Supplementary material for: “You left a void that we will never be able to fill”: The legacy of Edmond Amran El Maleh in a contemporary Moroccan novella
Source: Open Res Eur. 2023 Sep 29;3:165. [Version 1] doi: 10.12688/openreseurope.16430.1 (PMC10622859; doi:10.12688/openreseurope.16430.1)
Supplement: Peer review history

# Reviewer report: Reviewer response for version 1

**DOI:** 10.21956/openreseurope.17734.r35423

**Published:** 2023-11-02

**Authors:** Richard Ajah

**Affiliations:** 1 University of Uyo, Uyo, Akwa Ibom, Nigeria

The article studies Mohammed Said Hjiouij’s novella titled *Aḥǧiyat Idmūn ʿAmrān al-Māliḥ* published in 2020 and translated as *The Riddle of Edmond Amran El Maleh* as a narrative that hybridizes historical facts and fiction to produce a “faction”. The subject is highly innovating as it illustrates how literary writers use contemporary history as raw materials for literary production. All sections of the study are well structured and crafted in good English, showing the clarity of her arguments and the cogency of discursive engagements, laced with current literary and extraliterary bibliographic references on collective memory, trauma, Moroccan Jewish and Muslim identities, politics of un/belonging, cultural liminality among other thematic preoccupations.

 This study aligns itself with the traditions of textual and critical analysis. As a scholarly work, the author demonstrates a mastery of core concepts of postcolonial and postmodern theories as her discursive competence is unveiled in the postmodern reuse of the Moroccan Jewish scholar, Edmond Amran El Maleh as a springboard to interrogate the Moroccan Jewish nationality and its identity politics.  

 The relevance of this article is situated at its ability to problematize received grand narratives on the Moroccan Jewry and deconstruct its identity politics, thereby contributing to the cultural, historical, and social discourse of heterogenous Moroccan-ness.

Is the argument persuasive and supported by appropriate evidence?

Yes

Is the work clearly and cogently presented?

Yes

Is the topic of the essay discussed accurately in the context of the current literature?

Yes

Does the essay contribute to the cultural, historical, social understanding of the field?

Yes

Reviewer Expertise:

African (Maghrebi) literatures, Cultural studies, Migrant studies and Postcolonial theory

I confirm that I have read this submission and believe that I have an appropriate level of expertise to confirm that it is of an acceptable scientific standard.

# Reviewer report: Reviewer response for version 1

**DOI:** 10.21956/openreseurope.17734.r35418

**Published:** 2023-10-23

**Authors:** Cristina Dozio

**Affiliations:** 1 Università Statale di Milano, Milan, Italy

This articles tackles the memorialization of the Jewish community in Morocco and its departure since the late 1940s by examining the novella *The riddle of Edmond Amran El Maleh *(2020) by Mohammed Said Hjiouij. The first part provides a well documented historical overview of this process and a literature review. Then the second parts delves into the analysis of the novella on two levels: the positioning of non-conventional writings in the editorial market and the complex identity of Moroccan Jews inside and outside the country in comparison with other models of belonging.

 I have appreciated the focus on the novella's aesthetic features in connection with its main topic, the writer's interviews (conducted by the article's author or published in the press) and placing this issue in a Maghrebi/North African context in the post-independence and contemporary eras.

 Here I suggest some minor revisions or additions that the author might take into consideration:

1. Between the first and the second paragraph there is a comparison between the top-down and the bottom-up revival of the Jewish past; do the institutions support these creative endeavors or other cultural initiatives?
2. It is said that the third-person narrator embodies death; is this significant/symbolic? Some other passages mention that both protagonists narrate or tell their story; is the first-person narrator also used in the novella?
3. Have the real and fictional El Maleh ever visited Israel?
4. Is the sinking of Egoz mentioned by the writer a significant episode in the novella?  

Is the argument persuasive and supported by appropriate evidence?

Yes

Is the work clearly and cogently presented?

Yes

Is the topic of the essay discussed accurately in the context of the current literature?

Yes

Does the essay contribute to the cultural, historical, social understanding of the field?

Yes

Reviewer Expertise:

Contemporary Arabic literature, Moroccan prison writing, post-conflict writing, literary translation

I confirm that I have read this submission and believe that I have an appropriate level of expertise to confirm that it is of an acceptable scientific standard.
